# Supplementary material for: XYA-2: a marine-derived compound targeting apoptosis and multiple signaling pathways in pancreatic cancer
Source: PeerJ. 2024 Jan 16;12:e16805. doi: 10.7717/peerj.16805 (PMC10798151; doi:10.7717/peerj.16805)
Supplement: Supplemental Information 2 [file peerj-12-16805-s002.docx]

Supplemental file 1. Uncropped pictures of western blot

Mia PACA2 PANC-1

XYA-2 (μΜ) 0 5 10 20 0 5 10 20


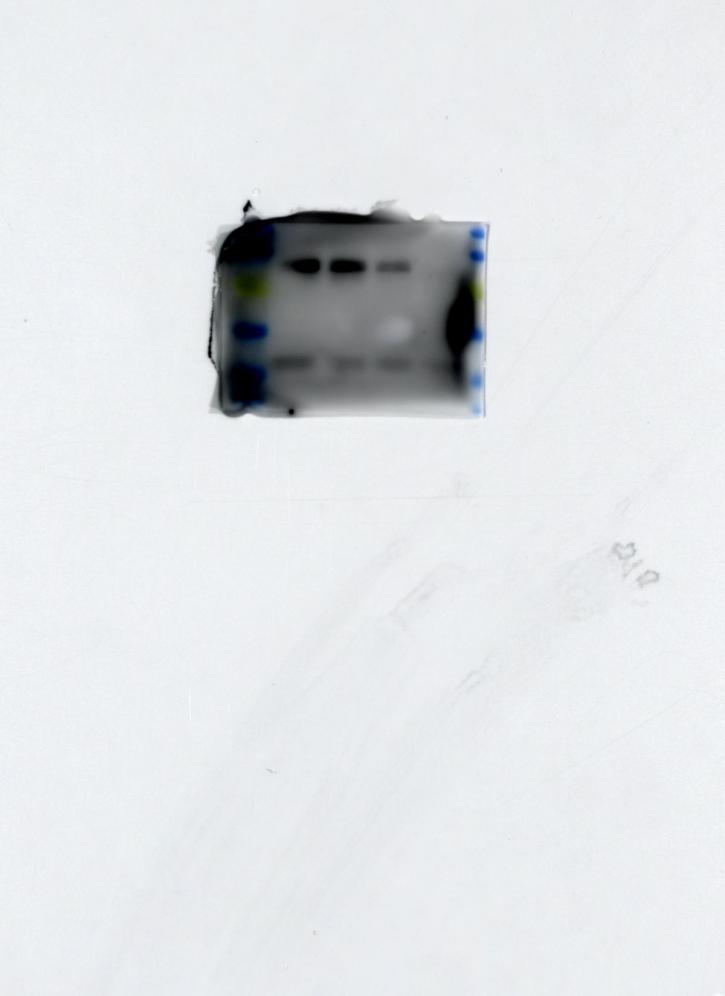

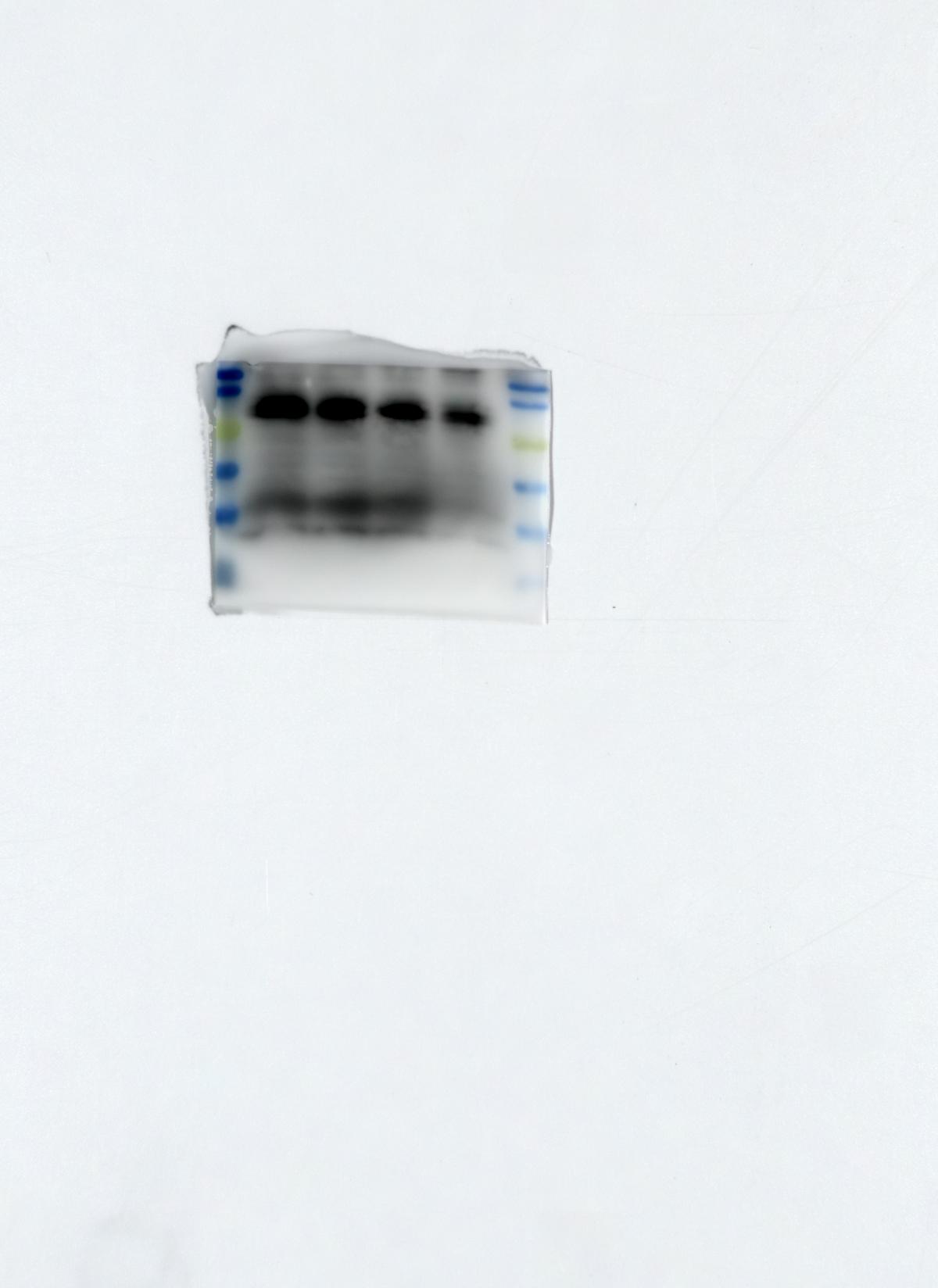
caspase 3 and cleaved caspase 3


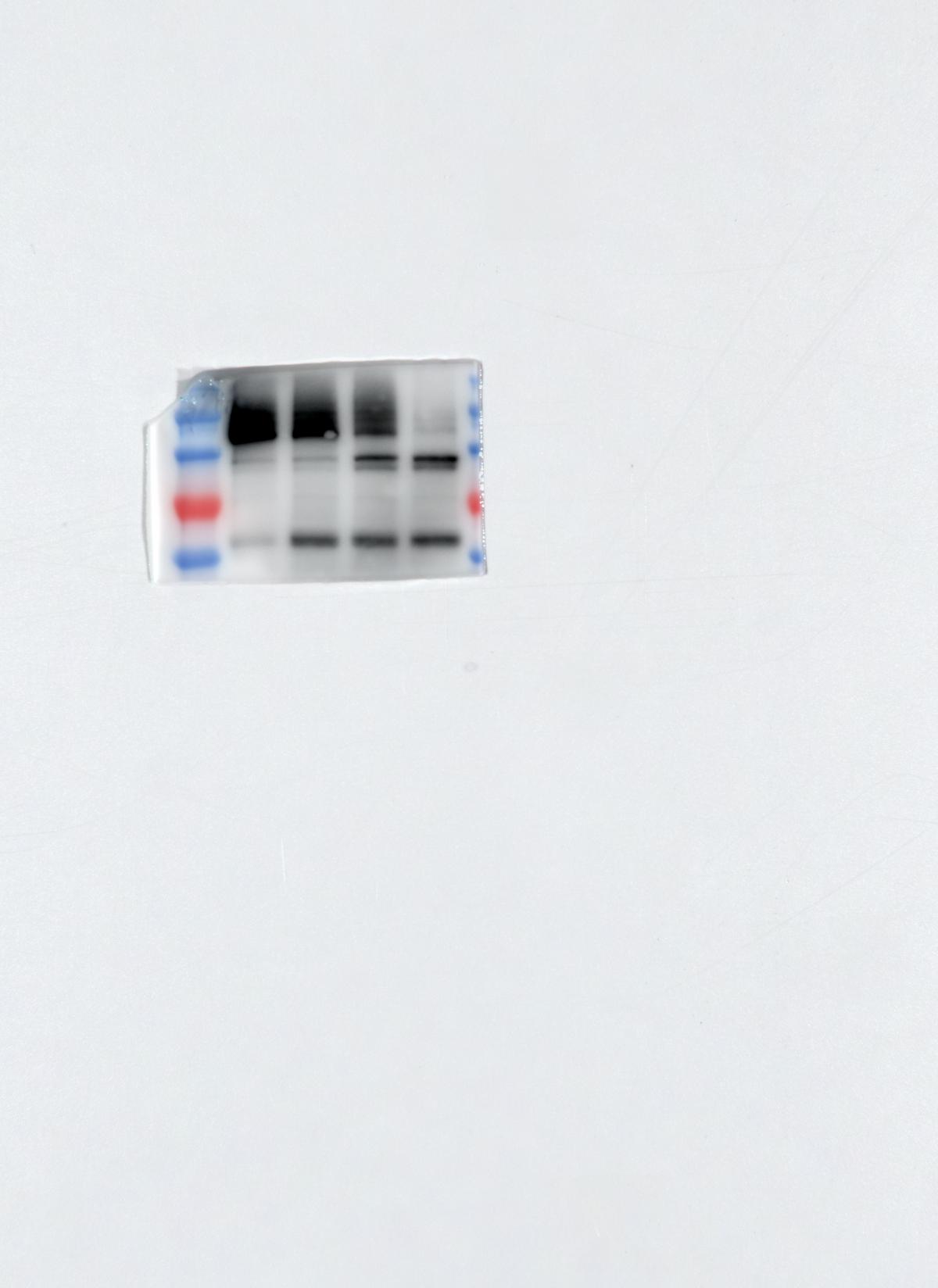

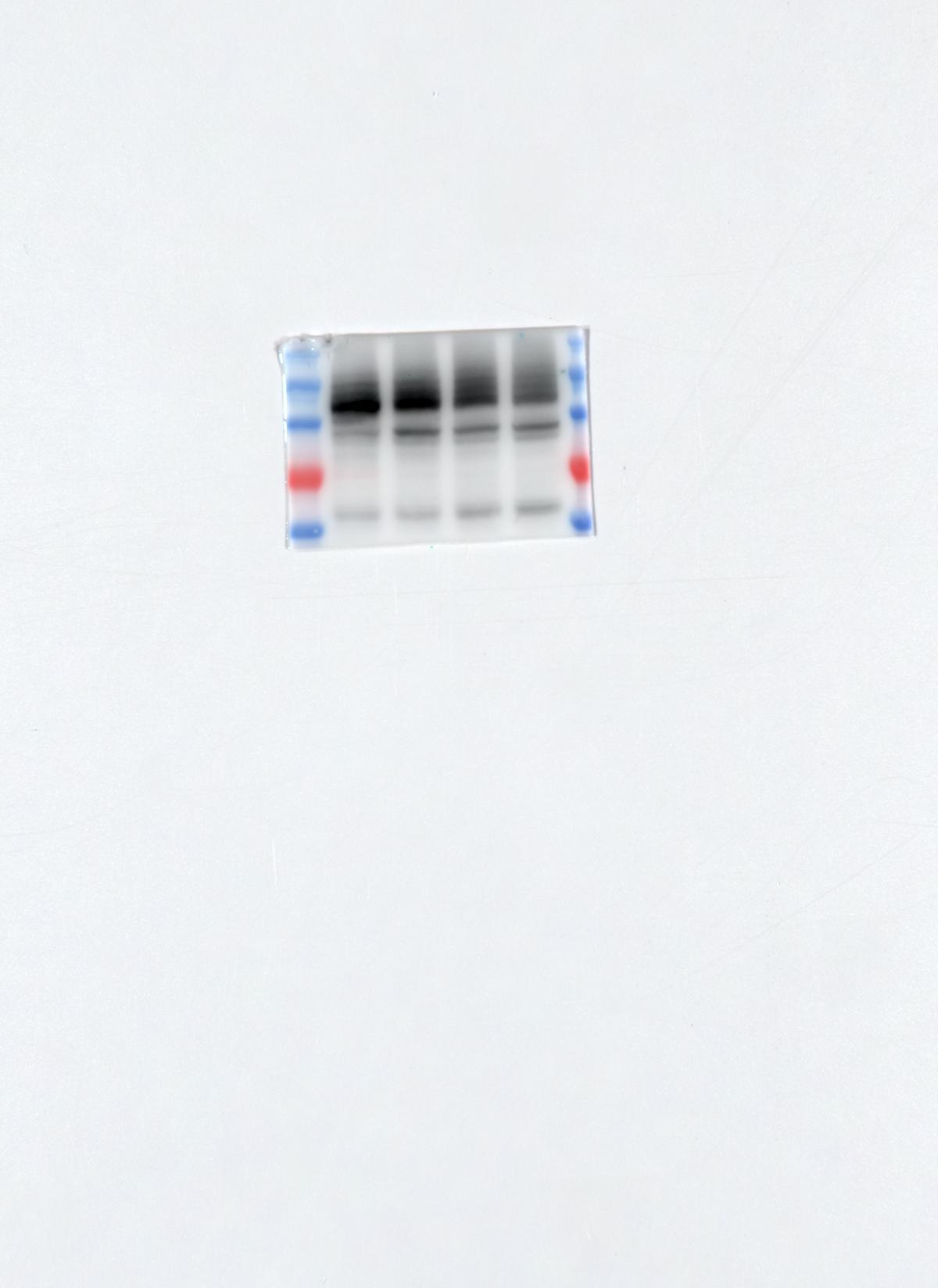
PARP


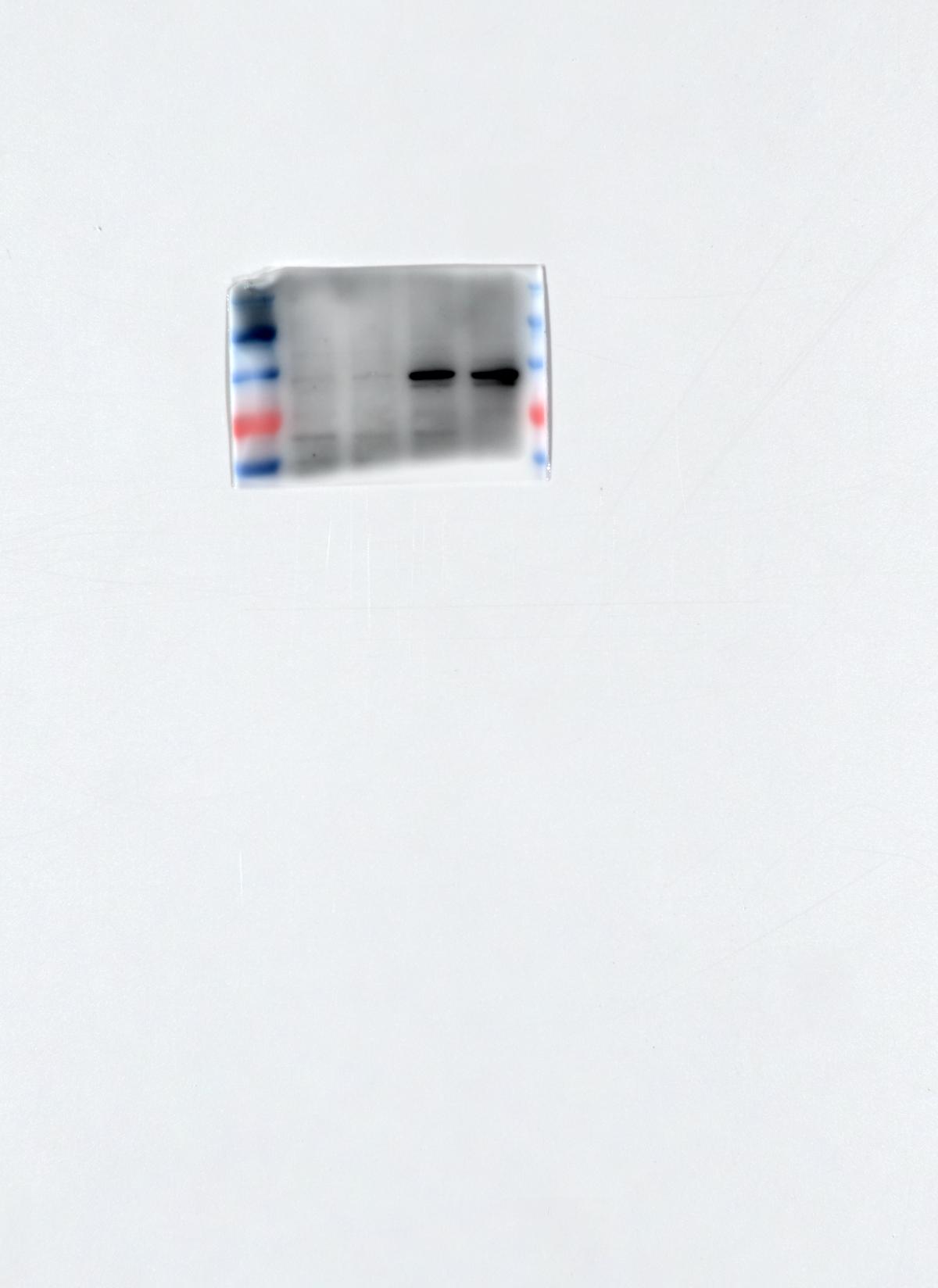

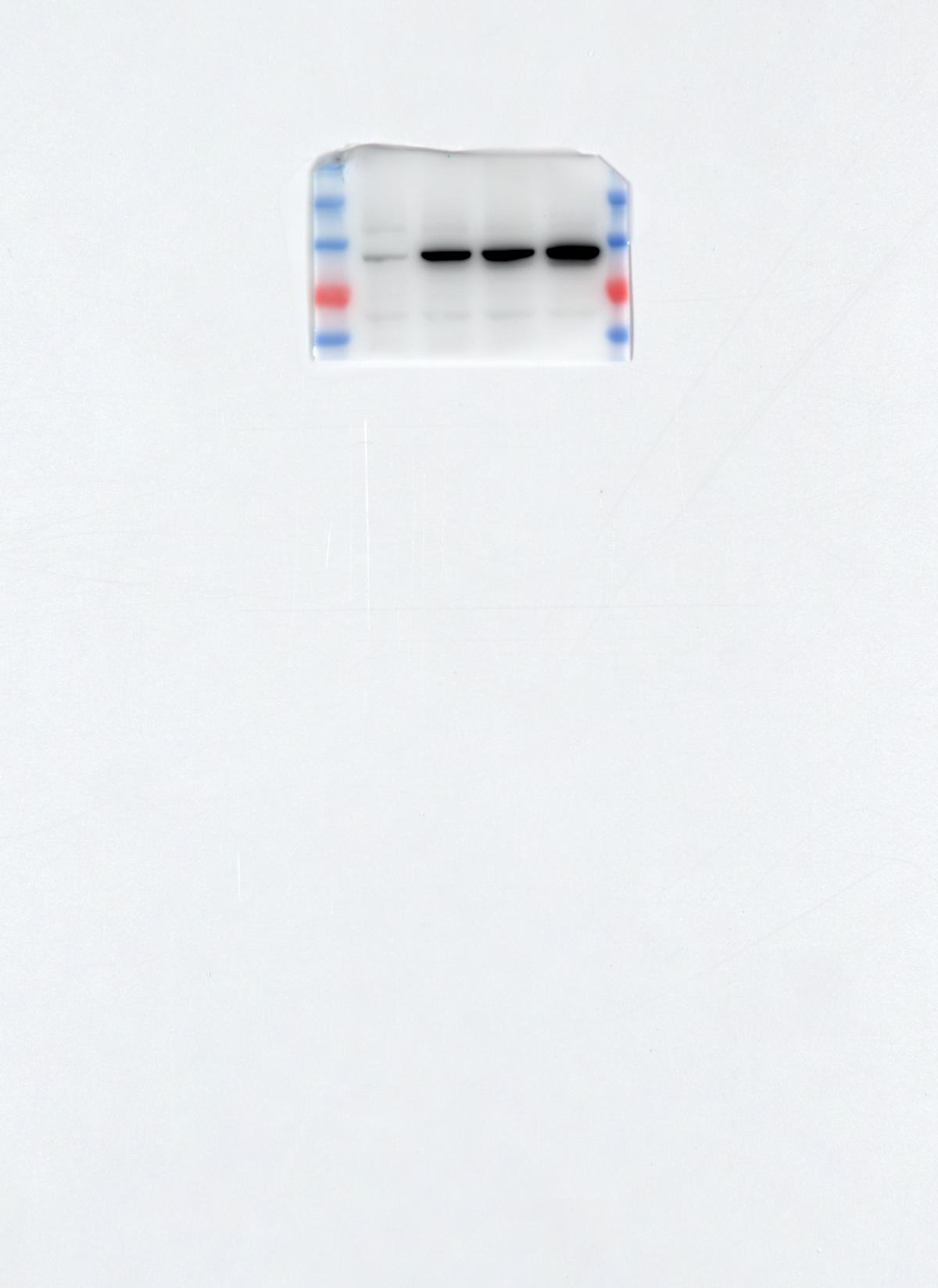
cleaved PARP


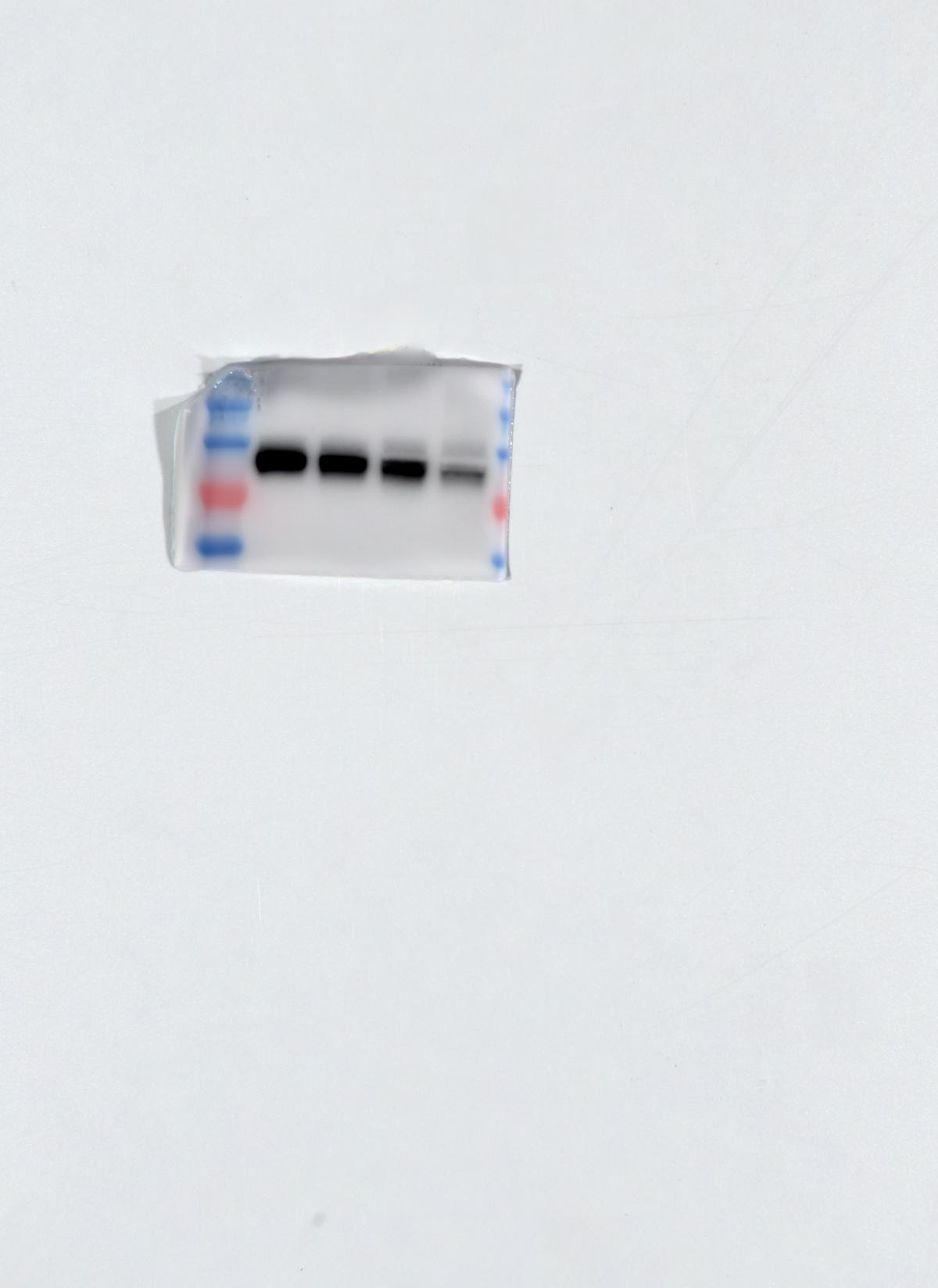

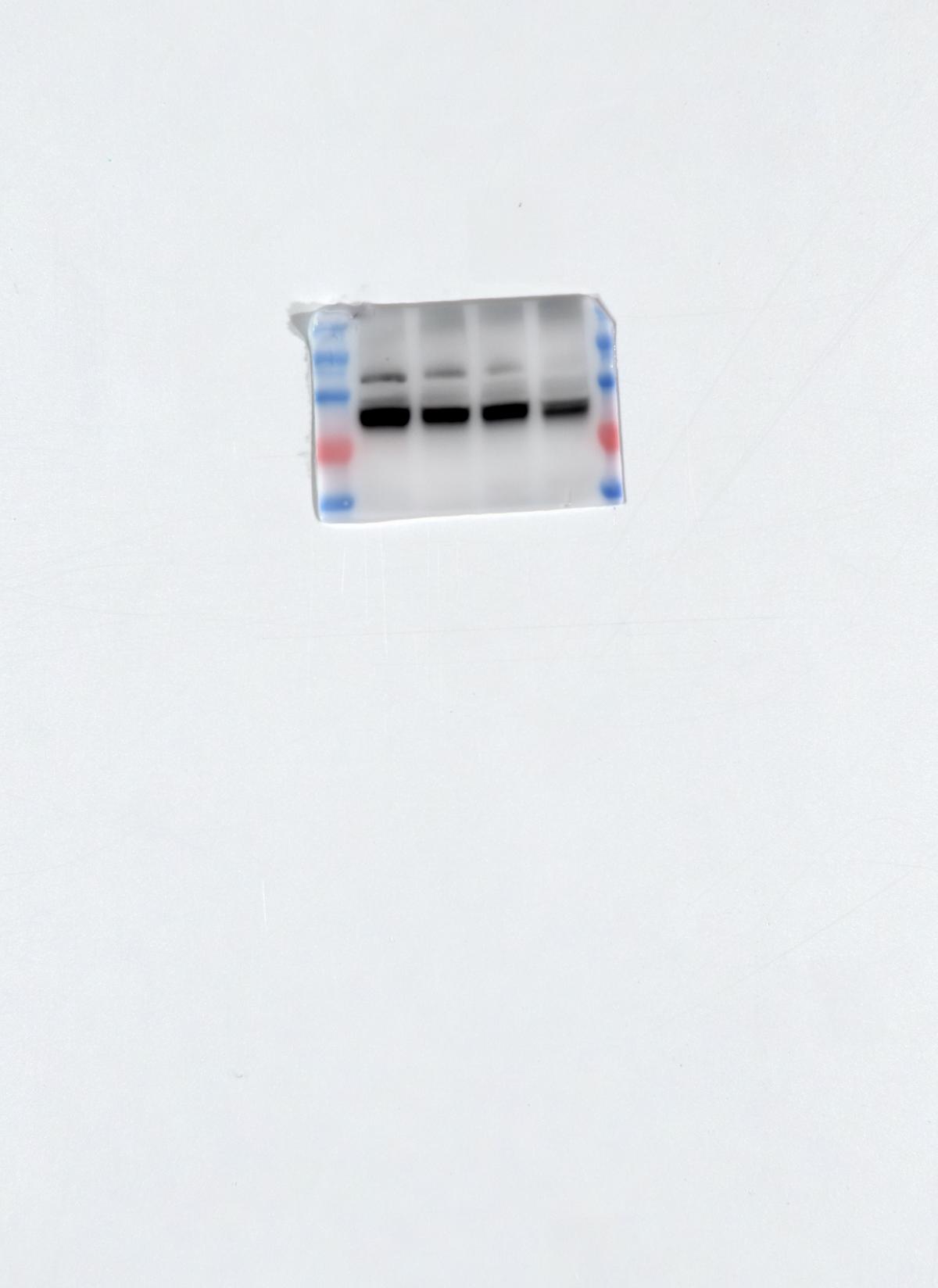
stat3


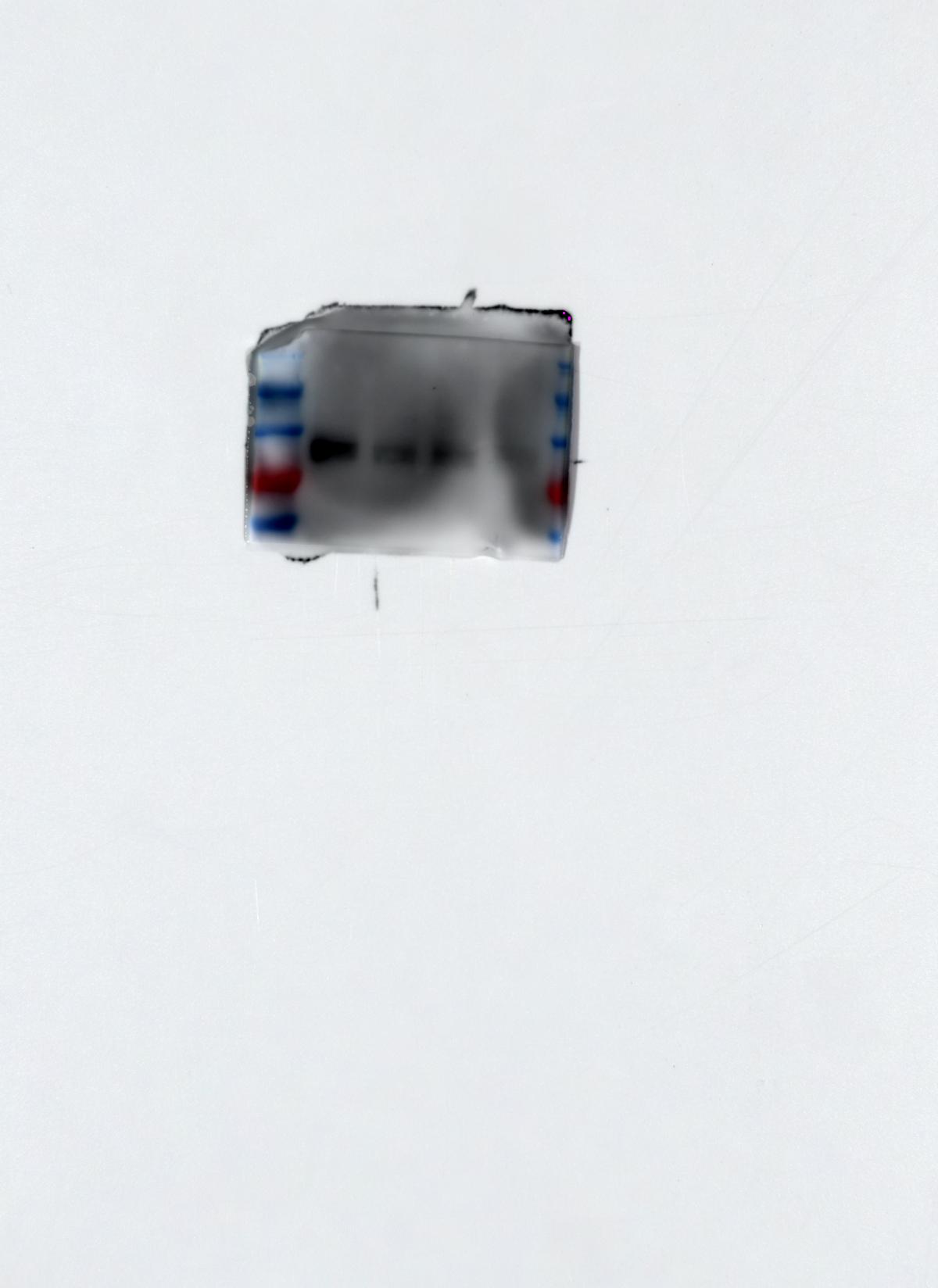

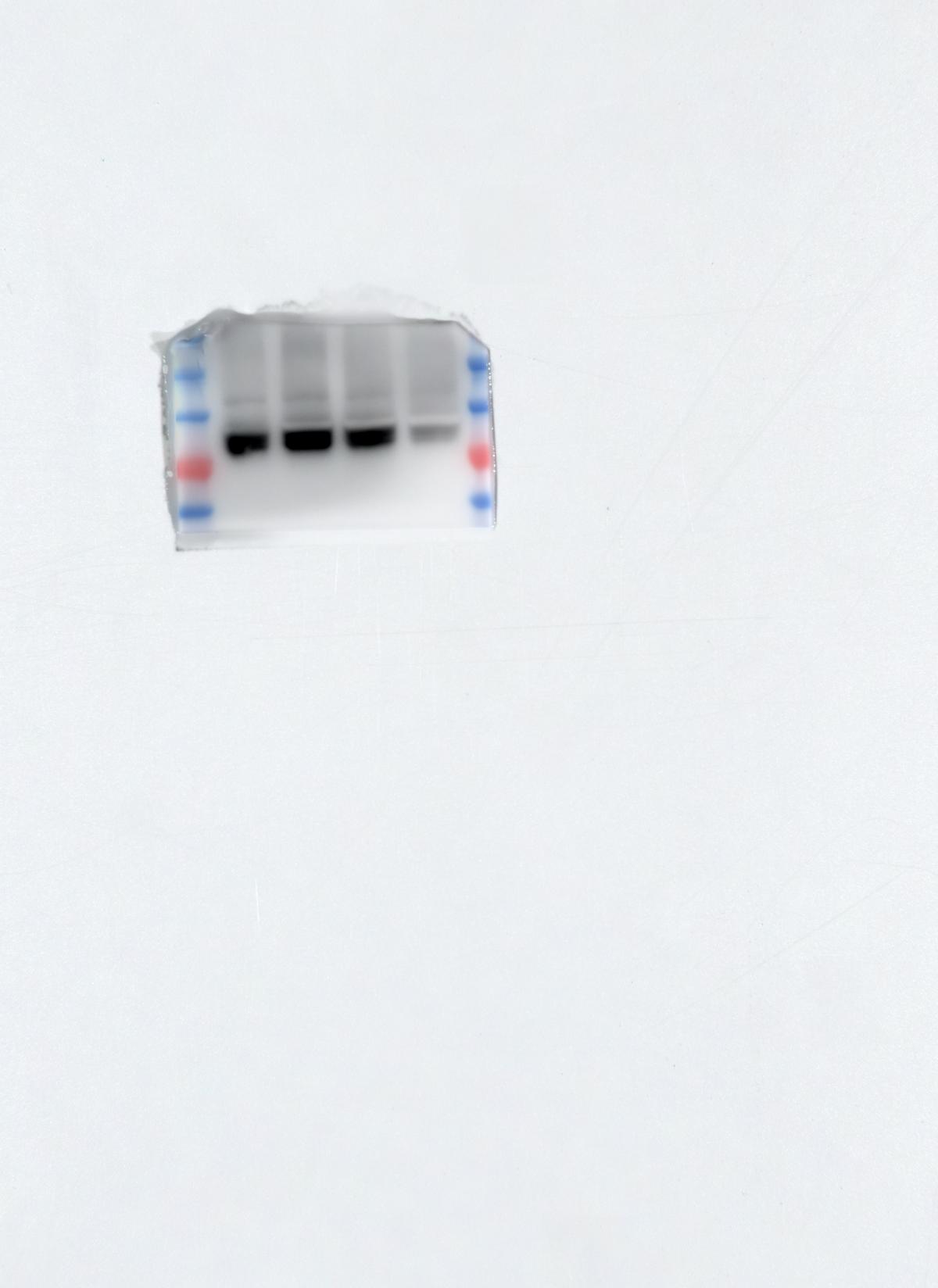
p-stat3(Y705)


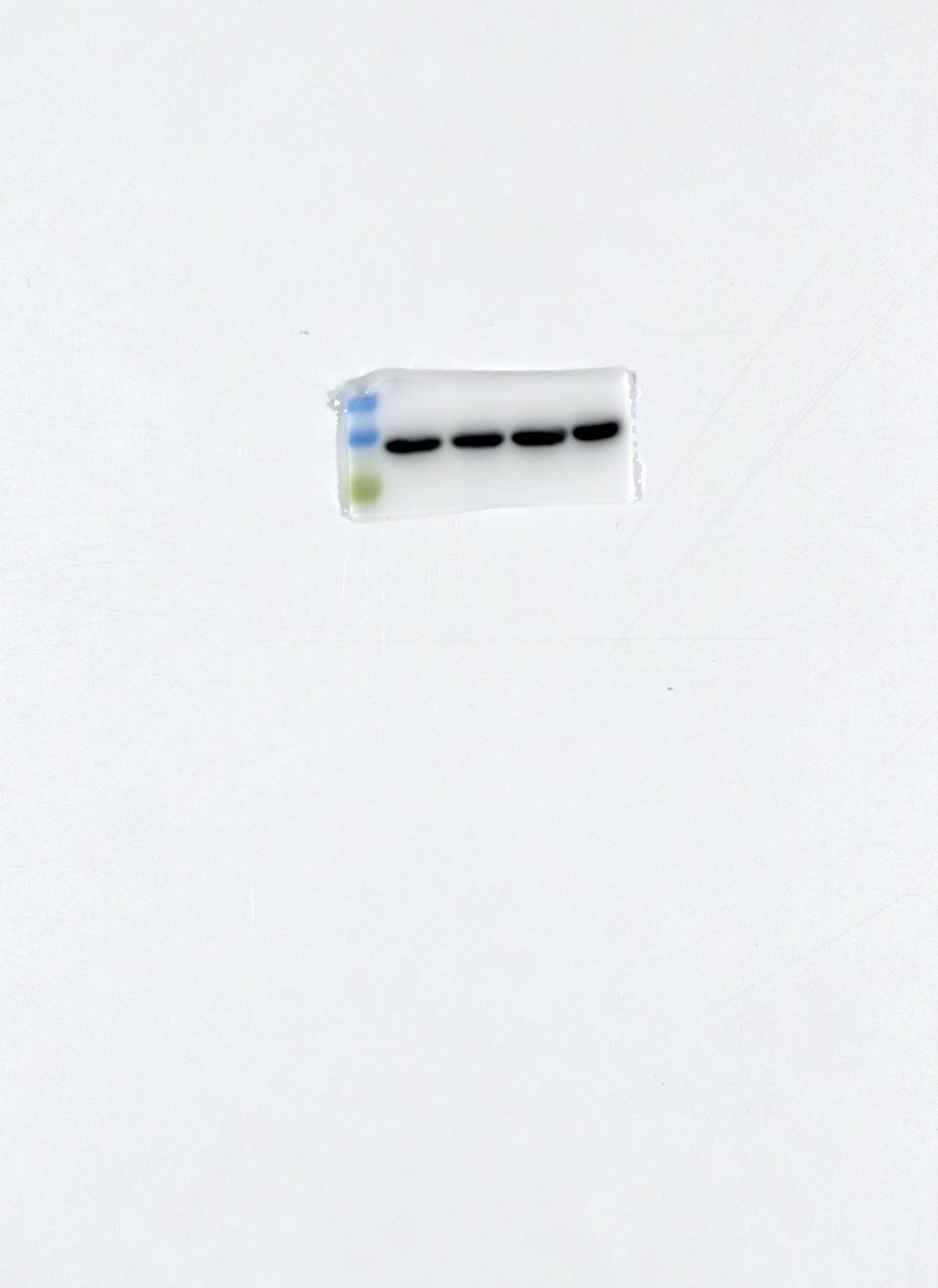

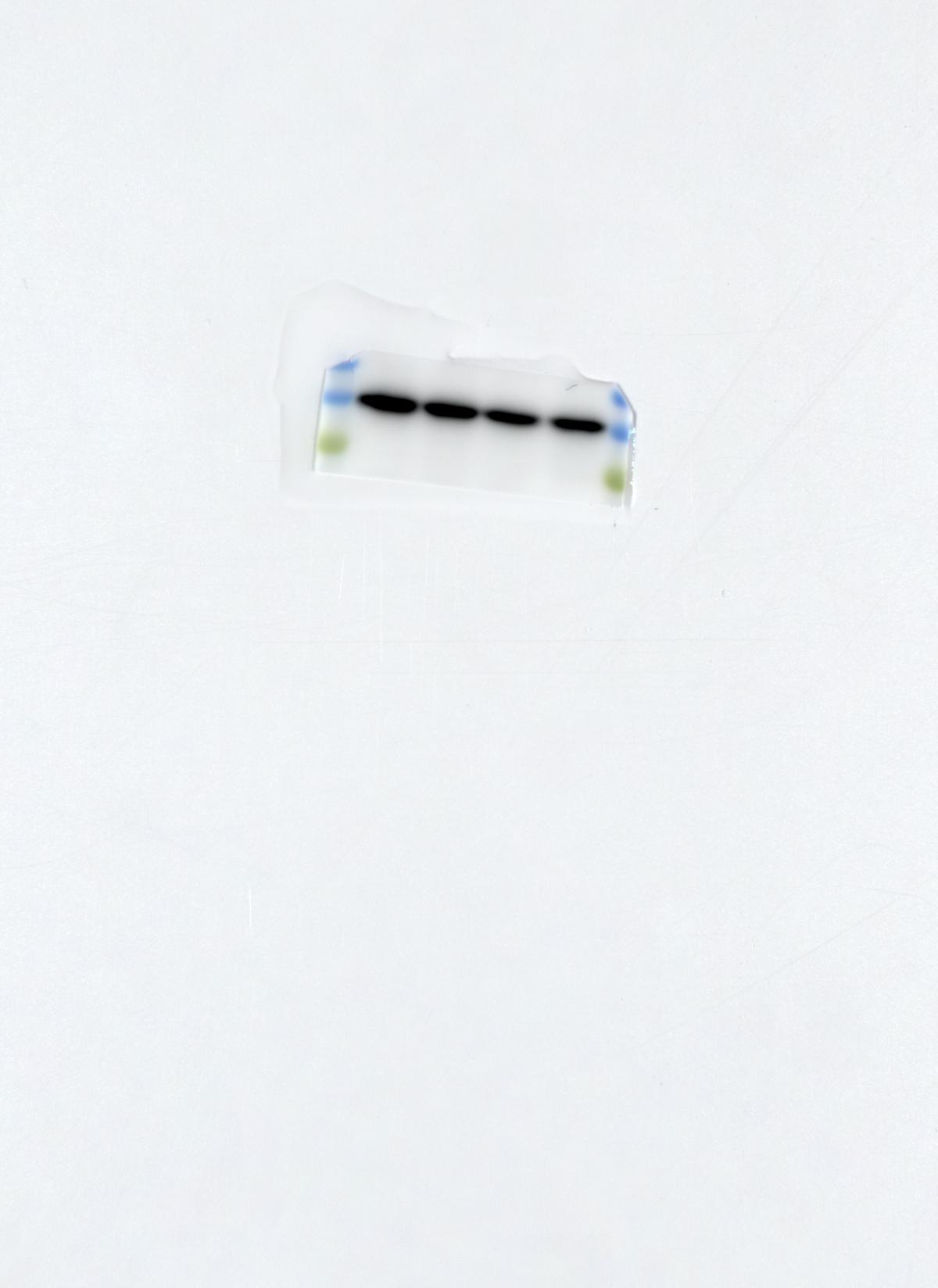
GAPDH
